# Central amygdala is related to the reduction of aggressive behavior by monosodium glutamate ingestion during the period of development in an ADHD model rat

**DOI:** 10.3389/fnut.2024.1356189

**Published:** 2024-05-03

**Authors:** Dewi Mustika, Yu Nishimura, Shinya Ueno, Shiori Tominaga, Takeshi Shimizu, Naoki Tajiri, Cha-Gyun Jung, Hideki Hida

**Affiliations:** ^1^Department of Neurophysiology and Brain Science, Nagoya City University Graduate School of Medical Sciences, Nagoya, Japan; ^2^Department of Physiology, Faculty of Medicine, Universitas Brawijaya, Malang, Indonesia; ^3^Department of Food and Nutrition, Shokei University Junior College, Kumamoto, Japan

**Keywords:** umami, c-Fos, aggression, resident-intruder test, gut-brain axis, vagus nerve, social isolation

## Abstract

**Introduction:**

Monosodium glutamate (MSG), an umami substance, stimulates the gut-brain axis communication via gut umami receptors and the subsequent vagus nerves. However, the brain mechanism underlying the effect of MSG ingestion during the developmental period on aggression has not yet been clarified. We first tried to establish new experimental conditions to be more appropriate for detailed analysis of the brain, and then investigated the effects of MSG ingestion on aggressive behavior during the developmental stage of an ADHD rat model.

**Methods:**

Long-Evans, WKY/Izm, SHR/Izm, and SHR-SP/Ezo were individually housed from postnatal day 25 for 5 weeks. Post-weaning social isolation (PWSI) was given to escalate aggressive behavior. The resident-intruder test, that is conducted during the subjective night, was used for a detailed analysis of aggression, including the frequency, duration, and latency of anogenital sniffing, aggressive grooming, and attack behavior. Immunohistochemistry of c-Fos expression was conducted in all strains to predict potential aggression-related brain areas. Finally, the most aggressive strain, SHR/Izm, a known model of attention-deficit hyperactivity disorder (ADHD), was used to investigate the effect of MSG ingestion (60 mM solution) on aggression, followed by c-Fos immunostaining in aggression-related areas. Bilateral subdiaphragmatic vagotomy was performed to verify the importance of gut-brain interactions in the effect of MSG.

**Results:**

The resident intruder test revealed that SHR/Izm rats were the most aggressive among the four strains for all aggression parameters tested. SHR/Izm rats also showed the highest number of c-Fos + cells in aggression-related brain areas, including the central amygdala (CeA). MSG ingestion significantly decreased the frequency and duration of aggressive grooming and attack behavior and increased the latency of attack behavior. Furthermore, MSG administration successfully increased c-Fos positive cell number in the intermediate nucleus of the solitary tract (iNTS), a terminal of the gastrointestinal sensory afferent fiber of the vagus nerve, and modulated c-Fos positive cells in the CeA. Interestingly, vagotomy diminished the MSG effects on aggression and c-Fos expression in the iNTS and CeA.

**Conclusion:**

MSG ingestion decreased PWSI-induced aggression in SHR/Izm, which was mediated by the vagus nerve related to the stimulation of iNTS and modulation of CeA activity.

## 1 Introduction

Developmental events, including the interplay of genetic, biological, environmental, and individual factors, can profoundly impact long-term emotional and behavioral responses (1–4). The long-term consequences of early life experiences play a crucial role in the onset of psychopathologies later in life (5–7). External environmental stimuli during development can affect the formation of emotions such as anxiety, antisocial behavior, and aggression (3, 8, 9). Aggression, a key symptom in various psychological disorders like mood disorders, personality disorders, substance abuse, schizophrenia, and autism, is often linked to early life environmental stressors such as social rejection or isolation in humans and other species (6, 10, 11). The impact of social isolation on aggressive behavior is contingent on the stage of development in rodents (10). Aggression levels increased in adult animals that underwent post-weaning social isolation (PWSI) (12, 13). PWSI disrupts the preference for social stimulus in adulthood and promotes the escalation of aggressive behaviors (7, 13–17).

The effect of social isolation stress during development on aggressive behavior largely depends on the strain, sex, and species of the animal model (10, 18, 19). The inbred juvenile spontaneously hypertensive rats (SHR) that exhibit hyperactivity, inattention, impulsivity, and learning deficits in various behavioral paradigms have been widely used as behavioral models of attention-deficit hyperactivity disorder (ADHD) (20, 21). Although stroke-prone SHR (SHR-SP) have been extensively studied as an ADHD model, no studies have reported which strain exhibits more aggressive behavior (22, 23). Impulsive aggression is a clinically distinct and common behavior in ADHD and autism, with 54% of ADHD patients showing clinical aggression (24). Individuals with ADHD tend to be more vulnerable to the impact of social deprivation (25, 26). Due to impulsivity and emotional dysregulation, aggression can be a challenging and complex issue for individuals with ADHD (25–29).

Food and nutrition are potential environmental modifications that may have an impact on behavior (29–32). The phenomenon by which dietary components stimulate the gastrointestinal tract, impact the brain, and ultimately determine behavior has recently gained scientific attention (33, 34). The gut-brain axis is known to play an important role in regulating brain function, and as a result, influences psychological and emotional stability (35, 36). L-glutamate, a savory umami taste substance, has the potential to play a role in gut-brain axis communication via activation of taste receptors and subsequent vagus nerve (37–40). Our previous data in SHR showed that monosodium glutamate (MSG) ingestion successfully decreased strong aggressive behavior in a social interaction test (41). This effect was mediated via the vagus nerve, as proven by diminishing the effect of decreasing aggression by MSG after vagotomy (41). However, the data appeared to vary depending on the experimental conditions, despite showing a significant difference between groups.

Therefore, in this study, we initially aimed to establish improved experimental conditions to investigate the detailed brain mechanism of reducing aggression due to MSG ingestion. Specifically, we substituted the social interaction test with the resident-intruder test and observed aggressive behavior under dim red light during subjective nights to align with the behaviors of nocturnal animals. Prior to this, we compared aggression levels in different rat strains, including SHR/Izm, SHR-SP/Ezo, WKY/Izm, a genetic control rat, and Long-Evans, a strain commonly used for studying aggression (20–22, 42, 43), to confirm the proper model of aggression with a neuropathological background of ADHD. Furthermore, we analyzed aggression-related brain areas in these strains by comparing neuronal activation based on c-Fos expression (44, 45).

Finally, the effect of MSG on aggression was investigated using the resident-intruder test in the most aggressive strain SHR/Izm. We validated the role of vagus nerve activation following MSG ingestion by conducting vagotomy and assessing neuronal activity in the nucleus of the solitary tract (NTS), which is the terminus of the vagus nerve’s gastrointestinal sensory afferent fibers (46). Since the brain mechanism underlying the effect of MSG ingestion has not been clarified yet, we focused on the alteration of neural activity in aggression-related areas of the brain, including the amygdala. The amygdala has been known to play a role in aggressive behavior and emotional instability in ADHD (47–50). The direct and indirect neuronal projections between the NTS and central amygdala (CeA) could lead to the hypothesis that NTS activation by MSG via the vagus nerve may potentially modulate neuronal activity in the CeA, ultimately affecting aggression (51–54).

## 2 Materials and methods

### 2.1 Animals

For experiments in strain-difference, male rats of four different strains (SHR/Izm, and SHR-SP/Ezo, WKY/Izm, and Long-Evans; *n* = 6/each strain) were purchased from Japan SLC Inc. (Hamamatsu, Japan). SHR/Izm represents an animal model of ADHD characterized by hyperactivity and inattention without anxiety-related impulsive behavior when compared to WKY (Wistar-Kyoto) rats as a genetic control (55, 56). SHR-SP is a sub-strain of SHR that exhibits more inattention and impulsivity than SHR (22, 57). Long-Evans was included for comparison, as it is a commonly used laboratory rat in studies aggression related to social isolation and intermale aggression (42, 43, 58, 59).

The most aggressive strain, SHR/Izm, was used to investigate the effects of MSG ingestion (60 mM solution) on aggression. A total of 24 male SHR/Izm were assigned to two groups: MSG group (*n* = 12) and control group (*n* = 12). Male Wistar rats (*n* = 12) were obtained from Japan SLC Inc. and used as intruders to induce aggression.

The rats were housed under temperature-controlled conditions (23-25°C, average humidity 50%) with free access to standard chow (MFG; Oriental Yeast Co. Ltd.) and water on a 12-h light/dark cycle (lights on at 22:00 and off at 10:00). To escalate aggression, animals were housed individually from P25 to P60 for 5 weeks in standard cages (40 × 23 × 18 cm) under conditions of low wood shaving bedding and relatively high illumination in the light phase. After 5 weeks of isolation, behavioral assessments, including the open-field test and resident-intruder test, were conducted in the dark phase at 13:00–16:00 to get more stable results. Intruder rats were group-housed (2–3 males per cage) in standard cages on a 12 h light/dark cycle (lights on at 08.00 and off at 20:00). MSG 60 mM solution (MP Biomedicals, United States, 101800) or distilled water was administered via drinking bottles *ad libitum* until behavioral tests were completed and the brains were obtained for c-Fos immunostaining. The drinking bottle was changed three times per week. Body weight and drinking volume were measured three times weekly.

To confirm the MSG action on c-Fos expression in the brain without behavioral effect of the resident intruder test, 18 male SHR/Izm at 8 weeks-old that were group-housed (2–3 males per cage) in standard cages on a 12 h light/dark cycle (lights on at 08.00 and off at 20:00) were assigned to three groups (60 mM MSG group, *n* = 6; 180 mM MSG group, *n* = 6; and control group, *n* = 6) and then MSG was administered by gavage one shot.

Additional experiments were conducted for 5 weeks to confirm the effect of MSG ingestion without behavioral effects and to clarify the effects of PWSI. Twelve male SHR/Izm rats were assigned to three groups: control group-housed group (*n* = 4), control PWSI group (*n* = 4), and MSG PWSI group (*n* = 4). MSG 60 mM solution or distilled water was administered *ad libitum* via drinking bottles.

Every effort was made to minimize suffering and the number of animals used. All experimental procedures were approved by the Committee on Animal Experimentation of Nagoya City University Medical School, and were in accordance with the animal care guidelines of Nagoya City University.

### 2.2 Behavioral test

#### 2.2.1 Open field test

The rat at P60 was allowed to move freely for 10 min in a black circular arena (60 cm diameter × 50 cm height) in the dark phase (13:00–16:00) under red dim light (~2 lux) and recorded using an overhead camera for the following analysis (60). The recorded behavior was analyzed using an automated tracking system Smart software (Bio Research Center Inc., Nagoya, Japan) with the following parameters: (1) total distance moved, (2) duration of inactivity, (3) frequency of entrance into the center area, and (4) time spent in the center area (61, 62).

#### 2.2.2 Resident-intruder test

Aggressive behavior in several strains of rats, including Long-Evans, WKY/Izm, SHR/Izm, and SHR-SP/Ezo, was induced in the resident’s home cage by the intruder. The intruder is a male Wistar rat with a slightly smaller body weight (approximately 20 grams) compared to resident. Wood shaving bedding in the resident cage was kept for 1 week before the resident-intruder test. This test was performed three times in three consecutive days in the dark phase (13:00–16:00) under red dim light using the same resident and intruder pair.

Animal behavior was video-recorded using an overhead camera for 10 min and analyzed by measuring the frequency, duration, and latency of anogenital sniffing (weak aggression), aggressive grooming (moderate aggression), and attack behavior (strong aggression). Aggressive grooming is characterized by a lateral threat, upright posture, rearing or pouncing, and chasing. Attack behavior is defined as biting, clinch attack, and keeping down (63). Manual behavioral annotation and tracking were performed using Smart software (Bio Research Center Inc., Nagoya, Japan).

### 2.3 Immunohistochemistry of c-Fos

To determine the areas of the brain that are related to aggression and those that are affected by the administration of MSG, IHC of c-Fos was conducted to assess neuronal activity in the brain. Ninety minutes after the resident intruder test, the rats were deeply anesthetized with pentobarbital sodium (100 mg/kg, i.p.; Tokyo Kasei, Tokyo, Japan) and perfused transcardially with 0.1 M phosphate-buffered saline (PBS, pH 7.4) followed by 4% (w/v) paraformaldehyde (PFA, Sigma-Aldrich., St. Louis, United States) in PBS. The brains were post-fixed in 4% PFA overnight at 4°C, followed by 30% (w/v) sucrose until submerged. The brains were embedded in O.C.T. compound (Tissue-Tek, Sakura Finetek Japan Co., Ltd.) and frozen. Serial coronal sections (40 μm) were prepared using a cryostat (Leica CM 1520, Japan) and collected in a cryoprotectant (anti-frozen solution) containing 25% Ethylene Glycol and 25% Glycerol in PB before histological analysis.

For immunohistochemistry, the sections were first washed with PBS for 5 min three times and incubated with 0.6% (v/v) H_2_O_2_ (Wako., Tokyo Japan) dissolved in PBS + 0.1% (v/v) Triton X-100 (Nacalai Tesque, Inc., Kyoto, Japan) for 30 min to inactivate endogenous peroxidase, and then incubated in a blocking solution (10% horse serum in PBS containing 0.3% Triton X-100) for 60 min following washing (PBS-T: PBS + 0.3% Triton X-100) for 5 min three times.

The sections were incubated with mouse monoclonal anti-c-Fos antibody (1:1000; EnCor Biotechnology Inc., Florida, MCA 2H2, lot.030123). After washing with 1% horse serum in PBS-T three times, the sections were incubated for 120 min in 4.5 μL/mL of biotinylated anti-mouse IgG antibody (Vector Laboratories, Inc., California, BA-2000, lot.2B0622) diluted with 1% horse serum in PBS-T at room temperature. The sections were then washed three times in PBS-T, followed by incubation in 9 μL/mL of avidin-biotin complex (Vectastain ABC kit; Vector Laboratory, Inc., United States, PK4000, lot. 2 J1116) in PBS-T for 60 min. The sections were visualized with 0.25 mg/mL diaminobenzidine dissolved in PBS containing 0.009% H2O2 for 5–10 min at room temperature. The sections were mounted on gelatin-coated slides, air-dried, and gradually dehydrated using 50 to 100% ethanol. The brain sections were embedded in a cover slide.

Microscopic images were obtained using an Olympus AX70 microscope integrated with the U-PHOTO Universal Photo System. C-Fos positive cells (appearing as round shape and dark brown color) were manually counted in each region of interest (Paxinos and Watson Brain Map) using ImageJ software. The number of positive c-Fos cells was counted on both the left and right sides from four sections for each brain area in each rat.

### 2.4 Subdiaphragmatic vagotomy

Vagotomy was carried out at the sub-diaphragmatic level using SHR/Izm at P24, according to our previous report with some modifications (41). Briefly, after overnight food restrictions, the rats were anesthetized with an intraperitoneal injection of a 2 mL/kg mixture of medetomidine (0.185 mg/mL; Fujita Pharmaceutical Co., Ltd., Tokyo Japan), midazolam (1 mg/mL; Sando Co., Ltd., Tokyo Japan), and vetorphale (1.25 mg/mL: Meiji Animal Health Co. Ltd., Tokyo Japan). After a midline incision of the abdomen, the left lobes of the liver were moved aside and covered with saline gauze, and the stomach and lower esophagus were exteriorized from the peritoneal cavity and kept wet with saline. The dorsal and ventral trunks of the vagus nerve on the lower esophagus were cut at the subdiaphragmatic level using electrocauterization under a microscope. Subsequently, the organs were placed in the appropriate position, and the muscle and skin were tightly sutured. After the surgery, atipamezole hydrochloride (Orion Pharma, Expoo, Finland) was administered after completion of all surgical procedures at a dose rate of 0.4 mg/kg (i.p.). The rats were then returned to their home cages. Five weeks of individual housing and MSG administration via drinking bottles were performed before the behavioral test.

### 2.5 Statistical analysis

GraphPad Prism 9.0.0 software (GraphPad Software Inc.) was used for the statistical analysis. The resident-intruder test was analyzed using non-parametric analysis by Mann–Whitney or multiple comparisons Kruskal-Wallis followed by Dunn’s test. The open field test and immunohistochemistry data were analyzed using parametric analysis by unpaired t-test or one-way ANOVA, followed by Tukey’s multiple comparison test as a post-hoc test. A non-parametric analysis was conducted if the dataset did not follow a normal distribution. Differences were considered statistically significant at *p* < 0.05.

## 3 Results

### 3.1 Isolation-induced aggressive behavior in strain-dependent rats

**Figure 1:**
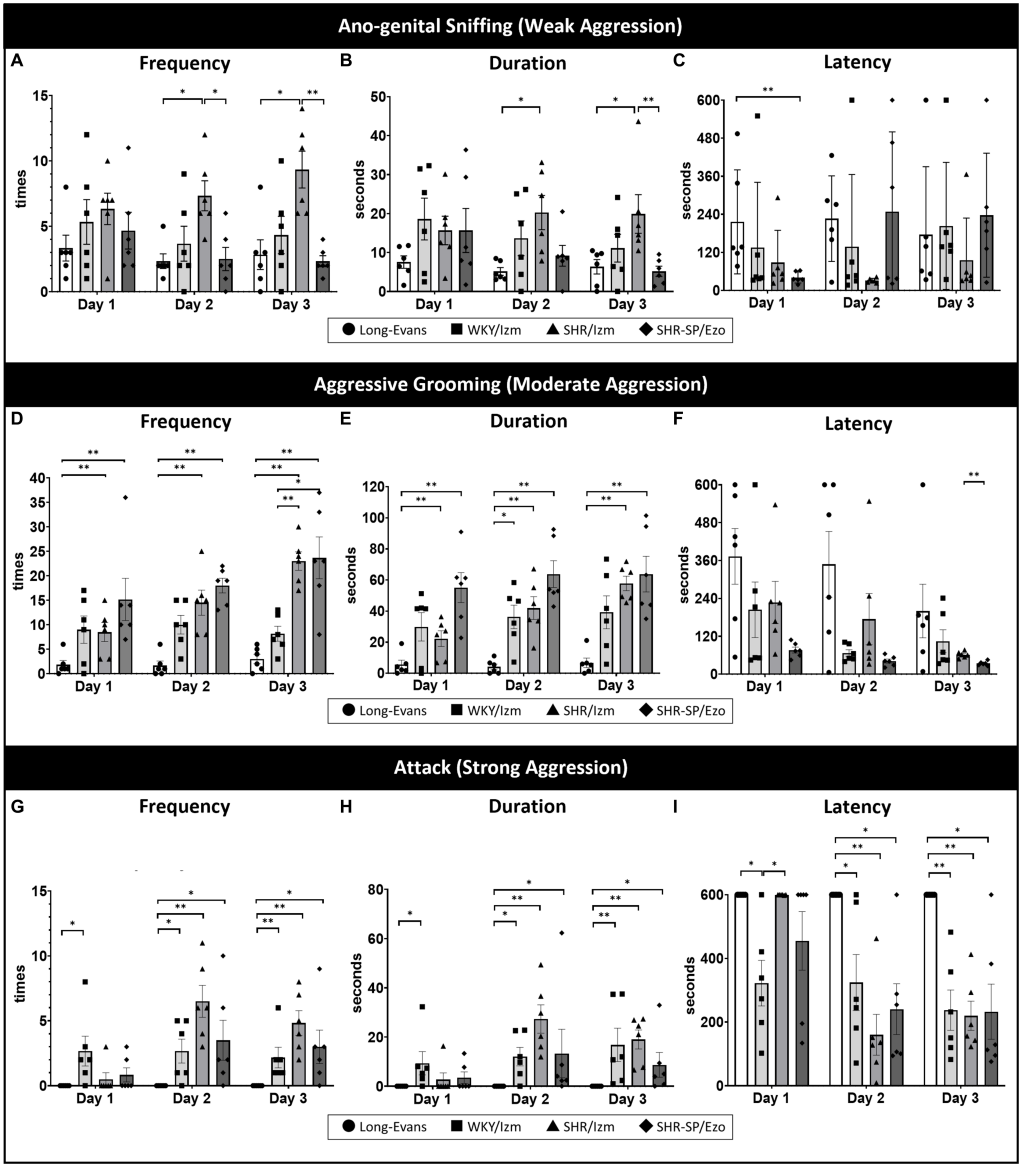
Isolation-induced aggressive behavior in strain-dependent rats was shown using a resident intruder test. The resident-intruder test was conducted to compare aggressive behavior among four different strains of rats: Long-Evans (*n* = 6), WKY/Izm (*n* = 6), SHR/Izm (*n* = 6), and SHR-SP/Ezo (*n* = 6) over three consecutive days. **(A–C)** SHR/Izm rats showed the highest frequency **(A)** and duration **(B)** of anogenital-sniffing (mild aggression) compared to other strains on days 2 and 3, even though there was no significant difference in the latency of anogenital-sniffing **(C)**. **(D–F)** High frequency **(D)** and long duration **(E)** of aggressive grooming (moderate aggression) were demonstrated by WKY/Izm, SHR/Izm, and SHR-SP/Ezo, whereas no difference in the latency of aggressive grooming was observed **(F)**. **(G,H)** SHR/Izm rats were the most aggressive strains, with the highest frequency **(G)**, longest duration **(H)**, and shortest attack behavior (strong aggression) **(I)**, showing a consistent significant difference compared with Long-Evans rats on three consecutive days of the resident-intruder test. Each bar represents the mean ± SEM (*n* = 6/each group); *p < 0.05, **p < 0.01, statistical analysis was performed using multiple comparisons (non-parametric test) using the Kruskal-Wallis test followed by Dunn’s test.

We previously reported that MSG ingestion, mediated by the vagus nerve, reduced aggressive behavior in the social interaction test (41). However, the data obtained from the social interaction test showed large variability, although significant behavioral differences were observed between the groups. Therefore, we first attempted to establish new experimental conditions that are more appropriate for a detailed analysis of the effect of MSG on the brain. Specifically, we substituted a social interaction test with a resident-intruder test conducted over three consecutive days, and observed aggressive behavior under dim red light during the subjective night, mimicking the conditions of nocturnal animals. We found that by providing relatively high illumination during the subjective day and conducting the resident-intruder test under dim red light during the subjective night, PWSI for 5 weeks from P25-P60 consistently and stably induced weak, moderate, and strong aggressive behavior (Figure 1).

After setting more appropriate conditions, we next compared aggressive behaviors across four different rat strains using the resident-intruder test: Long-Evans, WKY/Izm, SHR/Izm, and SHR-SP/Ezo (Figure 1). Although all strains exhibited anogenital sniffing (weak aggression) on the first day of the resident-intruder test (Figures 1A–C), we noticed an apparent enhancement in aggressive grooming (moderate aggression; Figures 1D–F) and attack (strong aggression; Figures 1G–I) day by day, with no difference between the second and third days. Among the four strains, Long-Evans was the calmest, exhibiting mild aggressive grooming with the lowest frequency, shortest duration, and longest latency (Figures 1D–F), without any attack behavior (Figures 1G–I). Conversely, SHR/Izm was the most aggressive strain, showing the highest frequency, longest duration, and shortest latency of attacks, especially on the second day (Figures 1G–I).

Thus, we revealed that only SHR/Izm showed significant differences from Long-Evans in all aggression parameters, including the frequency and duration of weak, moderate, and strong aggressive behavior.

### 3.2 Increasing c-Fos positive cells in the brain area associated with aggressive behavior

**Figure 2:**
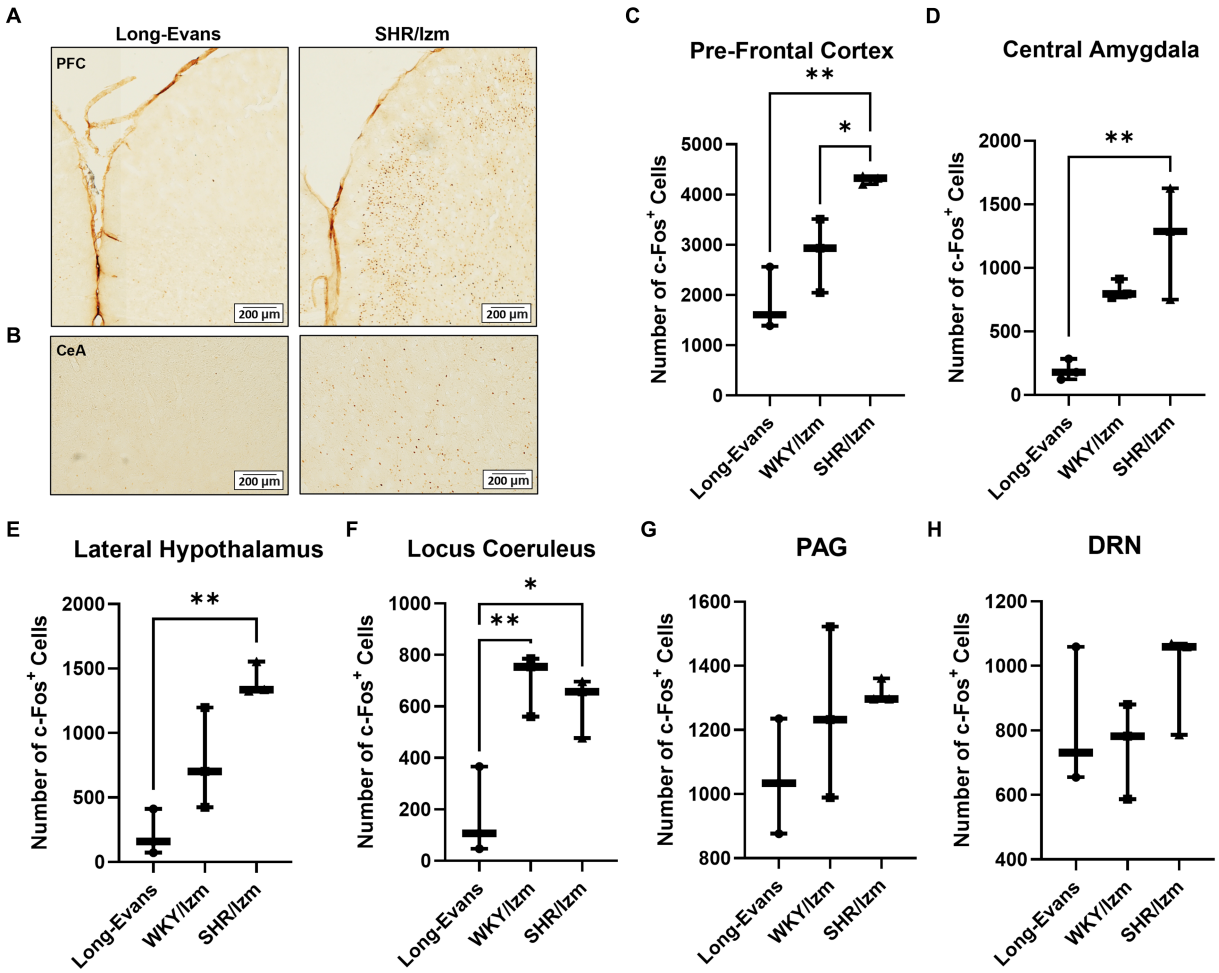
Increasing the number of c-Fos + cells in the brain areas associated with aggressive behavior. **(A,B)** Overview of c-Fos immunoreactivity in coronal sections of Pre-Frontal Cortex **(A)** and Central Amygdala **(B)** from Long-Evans rats and SHR/Izm rats as a result of IHC using anti c-Fos antibody. **(C–H)** Number of c-Fos + cells were counted from several areas of the brain related to aggression, including Pre-Frontal Cortex (PFC) **(C)**, Central Amygdala (CeA) **(D)**, Lateral Hypothalamus (LH) **(E)** Locus Coeruleus (LC) **(F)**, Peri-aquaductal Grey (PAG) **(G)**, and Dorso-Raphe Nuclei (DRN) **(H)** of Long-Evans (*n* = 3), WKY/Izm (*n* = 3) and SHR/Izm rats (*n* = 3). Increased c-Fos + cells were shown by SHR/Izm, especially significantly different compared with Long-Evans in the PFC, CeA, LH, and LC. Each bar represents the mean ± SEM (*n* = 6); *p < 0.05, **p < 0.01, statistical analysis was performed using one-way ANOVA followed by Tukey’s multiple comparison test.

Some brain areas are known to be related to aggressive behavior (18, 19, 64, 65). To investigate which brain areas are associated with strain differences in escalated aggression, IHC of c-Fos was performed in three different rat strains: Long-Evans, WKY/Izm, and SHR/Izm. The focus was on aggression-related brain areas such as the prefrontal cortex (PFC), central amygdala (CeA), lateral hypothalamus (LH), locus coeruleus (LC), periaqueductal gray (PAG), and dorsal raphe nucleus (DRN; Figure 2).

A higher number of c-Fos-positive cells was observed in the brains of WKY/Izm and SHR/Izm compared to the less aggressive strain rat, Long-Evans, especially in the PFC (Figures 2A,C), CeA (Figures 2B,D), LH (Figure 2E), and LC (Figure 2F). It is noted that the difference in c-Fos positive cells between Long-Evans and SHR/Izm was considerably large in the PFC (*p* = 0.0042), CeA (*p* = 0.0074), and LH (*p* = 0.0032; Figures 2C–E). However, no significant difference in c-Fos positive cells was detected in the PAG (Figure 2G) or DRN (Figure 2H) across the three rat strains.

### 3.3 Oral ingestion of MSG decreased isolation-induced aggression in SHR/Izm rats

**Figure 3:**
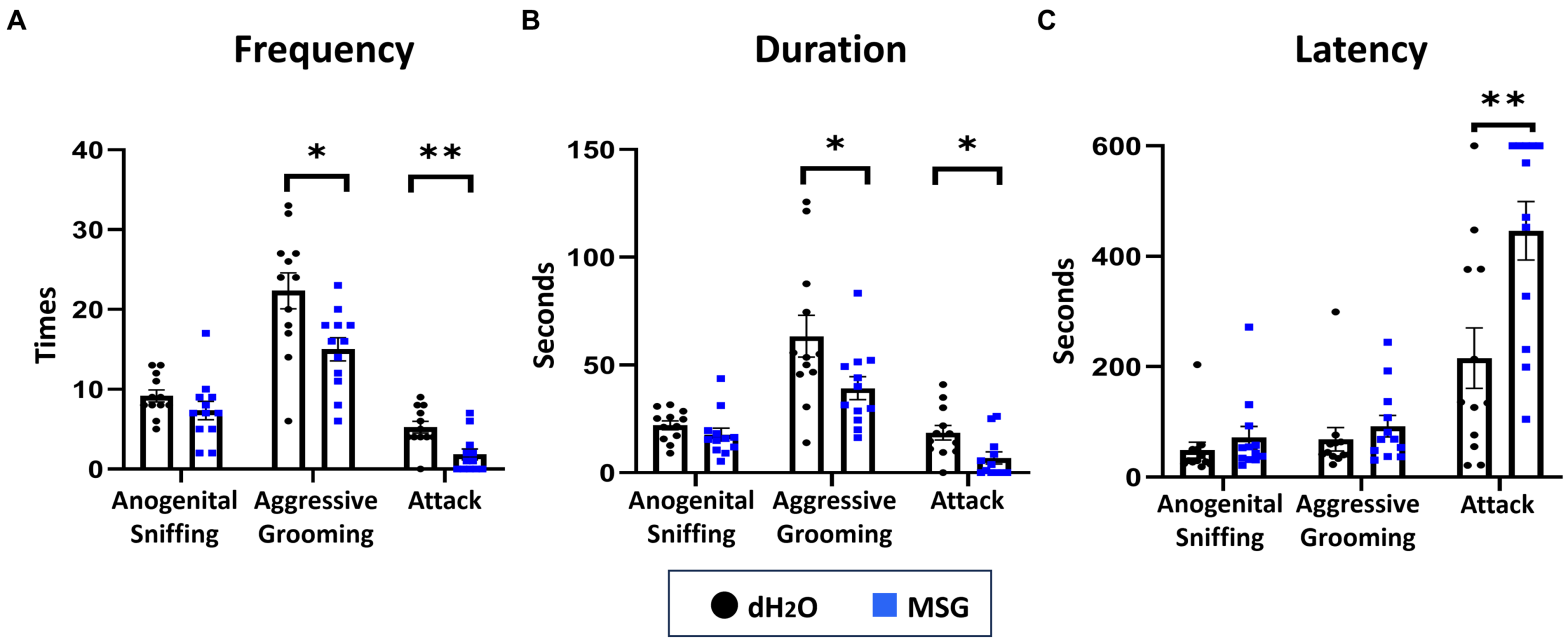
Oral ingestion of MSG decreased isolation-induced aggressive behavior in SHR/Izm rats was shown by the resident intruder test. The results of the resident intruder test showed that MSG ingestion significantly decreased the frequency of aggressive grooming and attack behavior **(A)**, decreased the duration of aggressive grooming and attack behavior **(B)**, and increased the latency of attack behavior **(C)** compared with the control group. Each bar represents the mean ± SEM (*n* = 12); *p < 0.05, **p < 0.01, statistical analysis was performed using Mann–Whitney test.

To confirm the effect of MSG in reducing aggression using our new experimental conditions, SHR/Izm rats were chosen based on previous data indicating that this strain is the most aggressive (Figure 1). Since the aggression on the second day of the resident-intruder test was obvious and consistent, we used data from the second day to compare the effect of MSG on aggression (Figure 3). MSG ingestion significantly decreased the frequency and duration of aggressive grooming (moderate aggression) and attack behavior (strong aggression; Figures 3A,B), and increased the latency of attack behavior (Figure 3C) compared to the control group that received drinking water without MSG.

**Figure 4:**
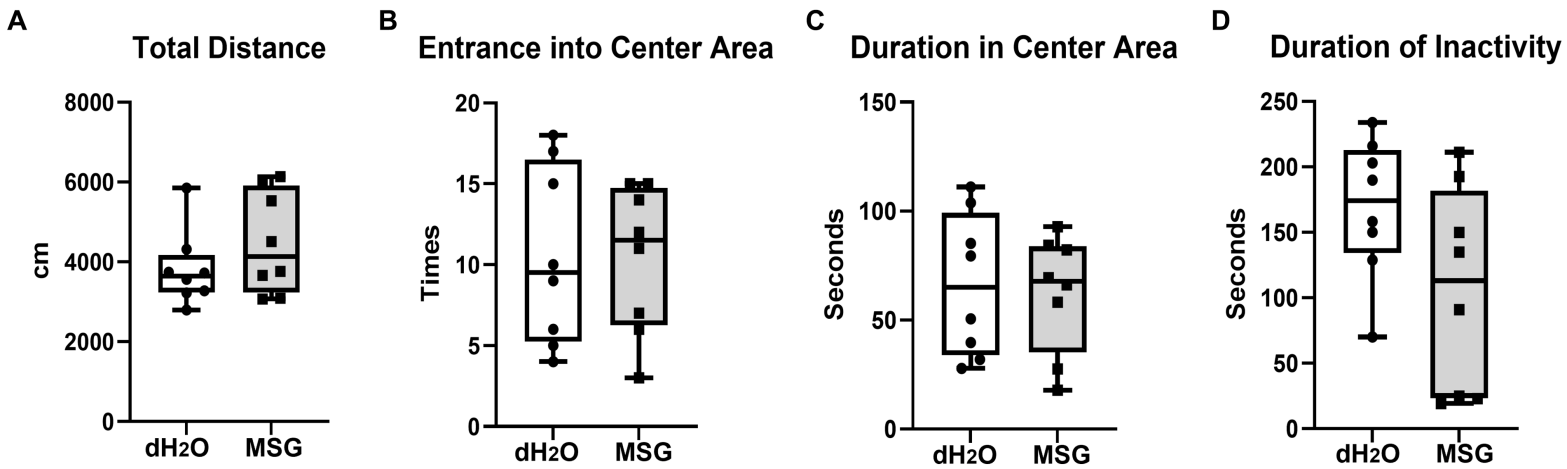
Oral ingestion of MSG did not affect the anxiety-like behaviors analyzed by open field test. The results of the open field test showed no difference between the MSG and control groups in the total distance traveled **(A)**, number of entrances into the center area **(B)**, time spent in the center area **(C)**, and total duration of inactivity **(D)**. Each bar represents the mean ± SEM (*n* = 8); statistical analysis was performed using unpaired t-test.

To confirm the effect of MSG on anxiety-like behavior under our new experimental conditions, an open field test was performed after 5 weeks of PWSI. No significant differences in the total distance traveled (Figure 4A), total number of entrances into the center area (Figure 4B), time spent in the center area (Figure 4C), and total duration of inactivity (Figure 4D) were observed between the MSG and control groups.

### 3.4 MSG administration increase c-Fos positive cells in the intermediate nucleus of solitary tract

**Figure 5:**
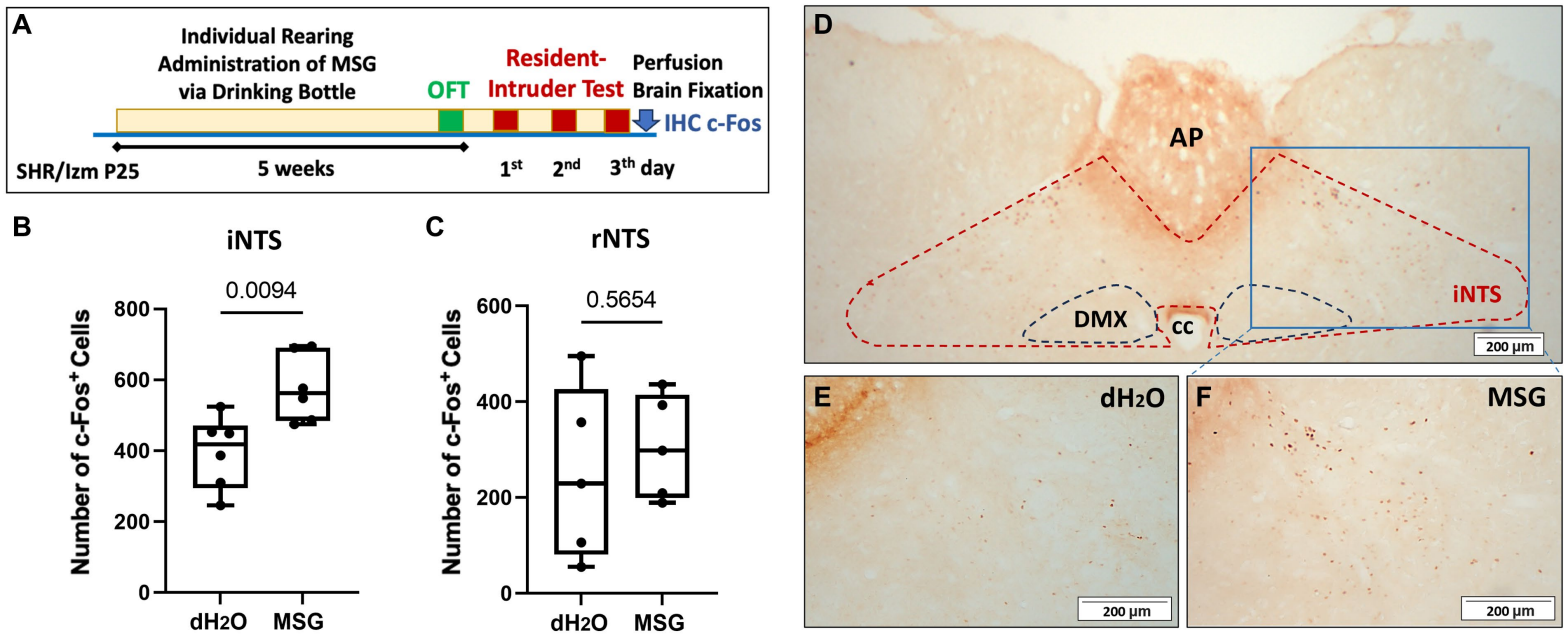
The effect of MSG ingestion on the number of c-Fos + cells in the nucleus of the solitary tract. **(A)** Schematic illustration of the experimental timeline. **(B,C)** Significantly increased c-Fos + cells were counted in the iNTS from the MSG group compared with the control group (*n* = 6/group) **(B)**, while no difference was observed in the rostral part of the NTS (rNTS) between both groups (*n* = 5/group) **(C)**. **(D–F)** Overview of c-Fos immunoreactivity in the coronal section of the intermediate part of the NTS (iNTS) **(D)** in the control **(E)** and MSG groups **(F)**. Each bar represents the mean ± SEM; statistical analysis was performed using unpaired t-test.

Our previous data showed that reduced aggression in the social interaction test was mediated by the vagus nerve (41). To confirm that the effect of MSG is related to the activation of the vagus nerve, which is connected to umami receptor stimulation in the gut, we performed c-Fos immunostaining in the nucleus of the solitary tract (NTS; Figure 5). We focused on both the intermediate part of the NTS (iNTS) as a terminal of the gastrointestinal sensory afferent fiber of the vagus nerve, and the rostral part of the NTS (rNTS) as a terminal of the tongue sensory afferent fiber of the glossopharyngeal nerve (46, 66).

Even though IHC staining was conducted after performing the resident intruder test without long-term fasting (Figure 5A), MSG ingestion significantly increased the number of c-Fos + cells in the iNTS (*p* = 0.0094; Figures 5B,D–F), whereas no difference was observed in the rNTS between both groups (Figure 5C).

### 3.5 The effect of MSG ingestion on c-Fos positive cells in the brain area associated with aggressive behavior

**Figure 6:**
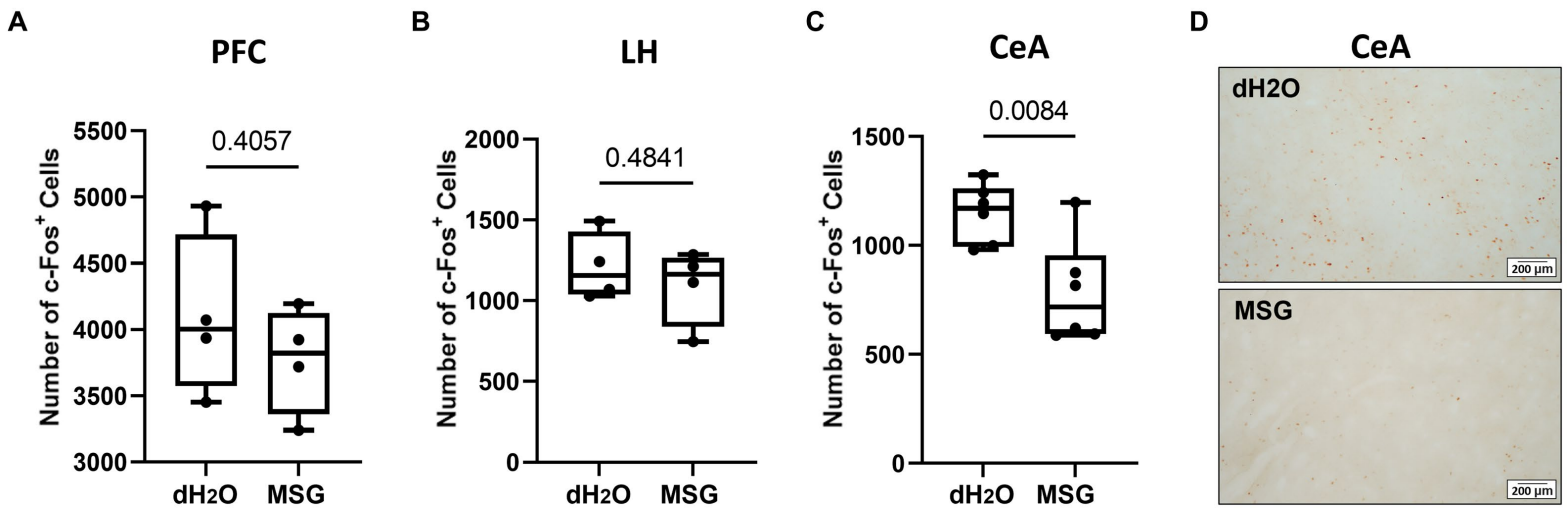
The effect of MSG ingestion on the number of c-Fos + cells in the PFC, LH, and CeA. **(A,B)** The number of c-Fos + cells was comparable in the PFC **(A)** and LH **(B)** between the MSG-ingested group (*n* = 4) and the control group (*n* = 4). **(C)** The number of c-Fos + cells decreased significantly in the CeA of MSG ingested group (*n* = 6) compared to that in the control group (*n* = 6). **(D)** Overview of c-Fos immunoreactivity in the coronal section of the CeA in the control and MSG groups. Each bar represents the mean ± SEM; statistical analysis was performed using Mann–Whitney Test.

We further investigated c-Fos expression patterns in aggression-related brain areas, such as the PFC, CeA, and LH, after MSG ingestion (Figure 6). The number of c-Fos + cells in the PFC and LH was comparable between the MSG-treated (*n* = 4) and control groups (*n* = 4; Figures 6A,B). However, the number of c-Fos + cells in the CeA was significantly lower in the MSG group than that in the control group (*p* = 0.0084; Figures 6C,D).

### 3.6 Intragastric administration of MSG increase c-Fos positive cells in the intermediate nucleus of solitary tract and central amygdala

**Figure 7:**
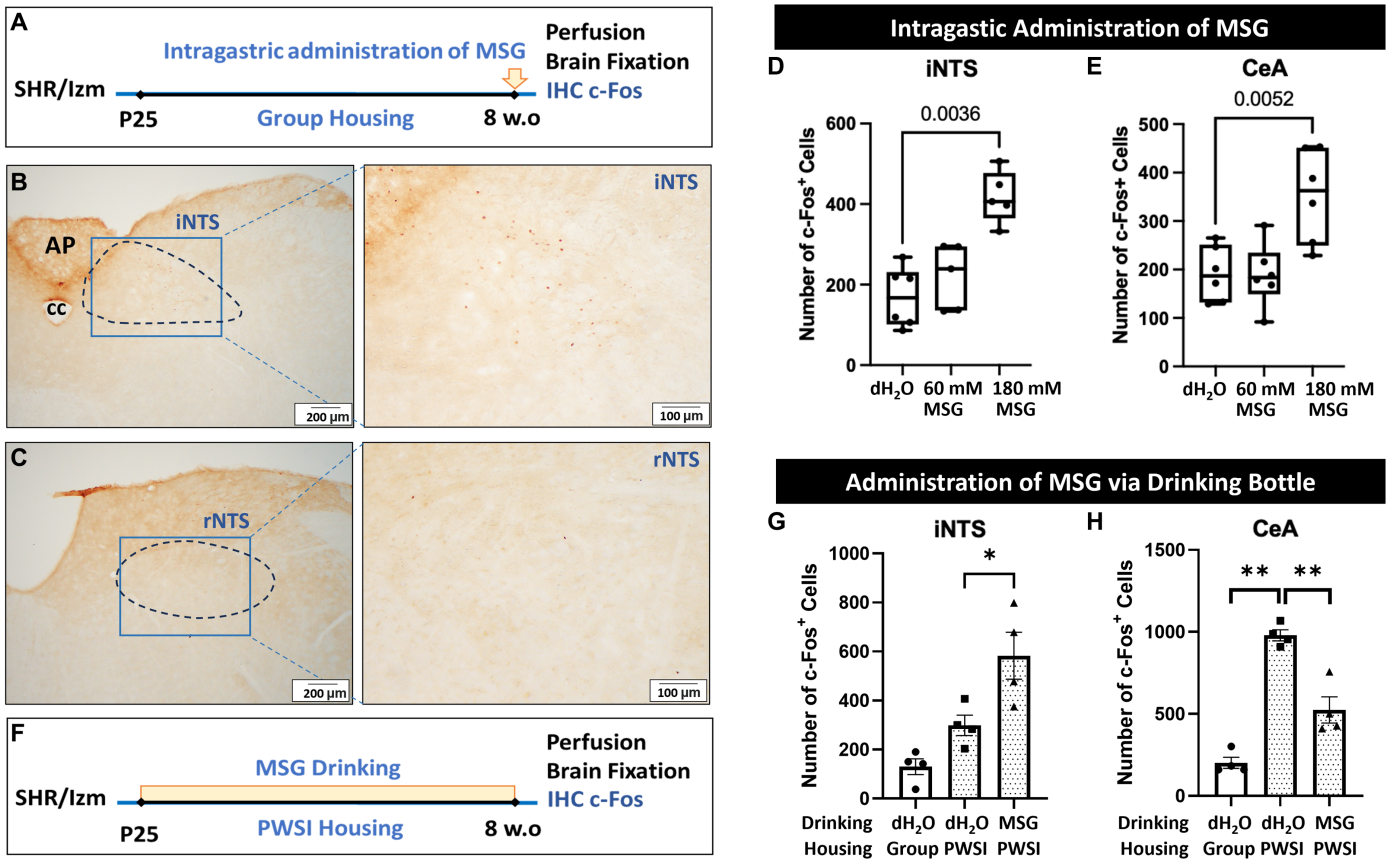
Intragastric administration of MSG increased the number of c-Fos + cells in the iNTS and CeA. **(A)** Schematic illustration of the experimental timeline for intragastric administration of MSG. **(B,C)** Overview of c-Fos immunoreactivity in the coronal sections of the iNTS **(B)** and rNTS **(C)**. **(D,E)** Intragastric administration of 180 mM MSG to SHR/Izm rats significantly increased the number of c-Fos + cells in the iNTS **(D)** and CeA **(E)**. **(F)** Schematic illustration of the experimental timeline for MSG administration via a drinking bottle. **(G)** The number of c-Fos + cells in the iNTS increased in the MSG drinking group compared to the control group. **(H)** The number of c-Fos + cells in the CeA was higher in the rats that experienced PWSI than in the rats in group housing, while MSG drinking reduced the number of c-Fos + cells elevated by PWSI. Each bar represents the mean ± SD (*n* = 4–6) and statistical analysis was performed using one-way ANOVA followed by Tukey’s multiple comparisons.

To confirm the direct action of MSG on c-Fos expression (without the effects of the resident intruder test and PWSI), we conducted another experiment without prior individual housing and resident intruder tests using male SHR/Izm at 8 weeks-old (Figure 7A). In this protocol, rats were directly administered MSG into the stomach after overnight fasting. Ninety minutes after MSG administration, c-Fos expression in the iNTS and rNTS was investigated.

We found that intragastric administration of 180 mM MSG resulted in a significant increase in c-Fos positive cells in the iNTS (Figures 7B,D). However, no positive cells were detected in the rNTS of the MSG-treated groups (Figure 7C), as in the control group.

Interestingly, in contrast to long-term MSG ingestion, acute direct administration of 180 mM MSG induced a significant increase in the number of c-Fos positive cells in the CeA compared with controls and 60 mM MSG-administration (Figure 7E). However, the total number of c-Fos + cells was considerably lower than in rats with prior behavioral tests and PWSI (Figure 6C).

To consider the possibility that PWSI affects c-Fos + cells, the number of positive cells in SHR/Izm rats grown in PWSI was investigated without behavioral tests (Figure 7F). We found that MSG ingestion increased the number of c-Fos + cells in the iNTS (Figure 7G). In addition, a significant increase in the number of c-Fos + cells was observed in the CeA of rats grown in the PWSI (Figure 7H). Interestingly, this enhanced expression in the CeA by PSWI was significantly reduced by ingestion of 60 mM MSG (Figure 7H), indicating that PWSI is a major factor that increases c-Fos + cells in the CeA, whereas MSG reduces the PWSI effect.

### 3.7 MSG effect on aggression and c-Fos expression in the iNTS and CeA was diminished by vagotomy

**Figure 8:**
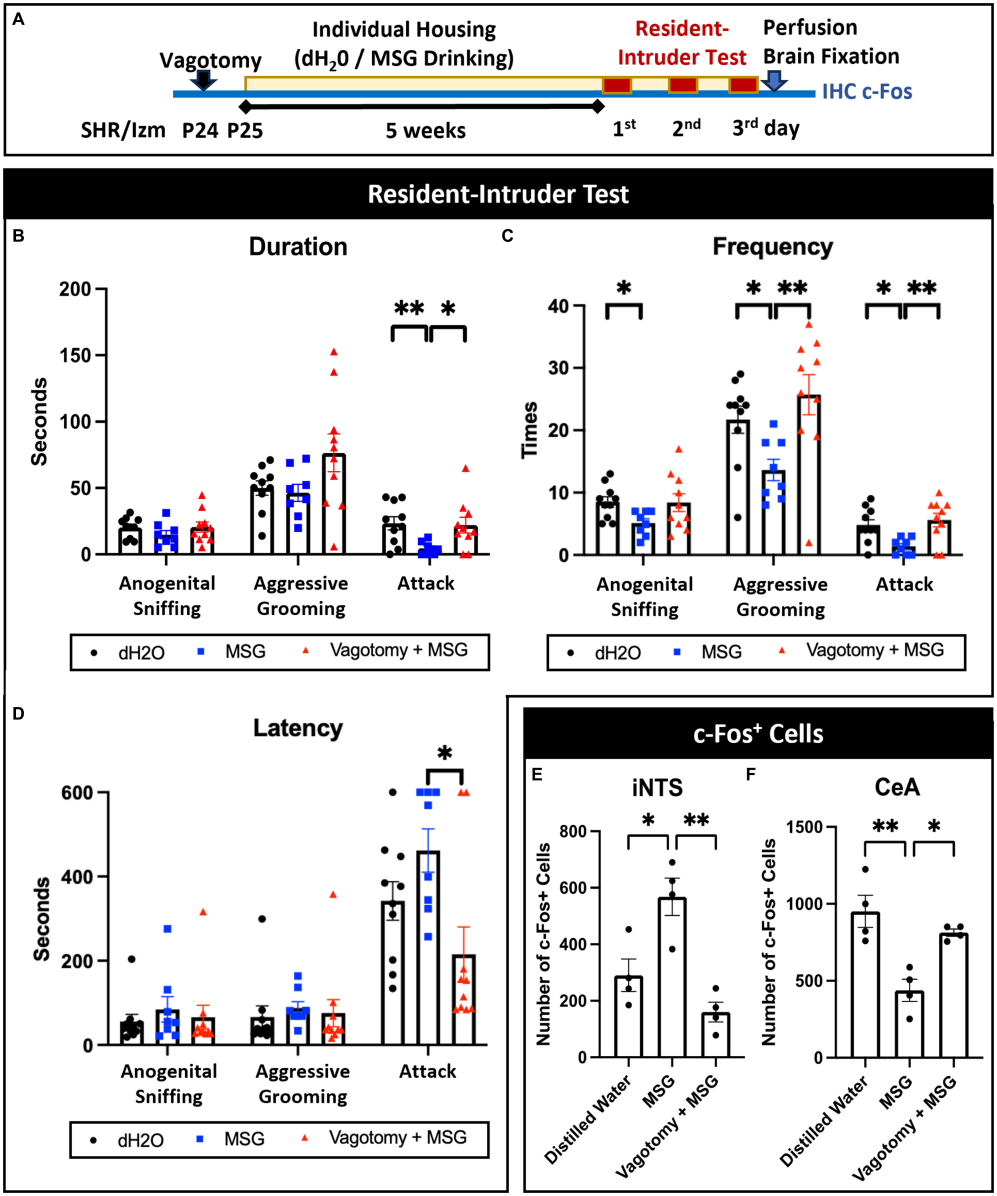
Vagotomy diminished the effect of MSG ingestion on aggressive behavior and c-Fos + cells in the iNTS and CeA. **(A)** Schematic illustration of the experimental timeline. **(B–D)** Vagotomy successfully diminished the effect of MSG on the duration of attack behavior **(B)**, frequency of aggressive grooming and attack behavior **(C)**, and latency of attack behavior **(D)**. **(E,F)** MSG ingestion significantly blocked the effect of MSG ingestion on the number of c-Fos + cells in the iNTS **(E)** and CeA **(F)**. Each bar represents the mean ± SEM; *p<0.05, **p<0.01, statistical analysis was performed using Kruskal-Wallis test followed by Dunn’s test for resident intruder test (*n* = 8) and one-way ANOVA followed by Tuckey’s multiple comparisons test for c-Fos + cells (*n* = 4).

Bilateral subdiaphragmatic vagotomy was performed at P23 to verify the importance of gut-brain interactions in the MSG effect (Figure 8A). Vagotomy blocked the effect of MSG on aggression in the resident intruder test; even with MSG ingestion, the duration of attack (Figure 8B), the frequency of aggressive grooming and attack (Figure 8C), and the latency of attack (Figure 8D) were similar to the control level. Vagotomy also diminished the effect of MSG on c-Fos expression in the iNTS (Figure 8E) and CeA (Figure 8F), indicating that the vagus nerve plays an important role in the effect of MSG on aggression and modulation of neuronal activity, not only in the iNTS but also in the CeA.

## 4 Discussion

In this study, after confirming that SHR/Izm is an appropriate model for aggression and identifying aggression-related brain areas, our hypothesis regarding how gut-brain stimulation by MSG potentially influences aggressive behavior was reaffirmed through the resident-intruder test and c-Fos immunostaining.

The level of aggression could vary depending on the rat strain, even though PWSI in SHR/Izm heightens aggression, as reported in Wistar rats and Sprague–Dawley rats (10, 13, 67, 68). SHR/Izm rats are often used as the animal model of ADHD although those lack impulsivity in several paradigms (22, 55, 69). SHR-SP/Ezo rats that were isolated as a SHR substrain, are known to have more impulsivity compared with SHR/Izm rats (70). As aggression is related to impulsivity in ADHD, we assumed that the SHR-SP/Ezo rats are potentially more aggressive. However, our data showed that SHR/Izm rats were more aggressive than the SHR-SP/Ezo rats. Higher motor activity in SHR/SP would be related to less aggression in the resident intruder test, since contact between the resident and intruder is relatively sorter due to the high movement of SHR-SP/Ezo (70). In contrast, Long-Evans rats displayed mild behavior without any attack behavior, despite undergoing the same PWSI conditions.

It is reported that PWSI affect the medial prefrontal cortex (mPFC) by decreasing dendritic density and glial cell number, inducing hyperactivity of glutamatergic and GABAergic neurons, and resulting impairment of excitatory/inhibitory balance (10, 12, 71). In optogenetic studies, the projection from the mPFC to the mediobasal hypothalamus contributes to the quantitative aspects of aggressive biting behavior, whereas the projection from the mPFC to the LH is linked to the qualitative aspects of abnormal aggressive behaviors (72). Therefore, the higher number of c-Fos positive cells in the PFC and LH of SHR/Izm compared to Long-Evans could explain the different aggression levels between these strains. Further studies are needed to understand the underlying mechanisms of PWSI-induced increased neuronal activity in the PFC and LH in aggressive rats, whereas less aggressive rats such as Long-Evans seem insusceptible to PWSI. Factors such as gene interactions and genetic background seem to play a role in the strain and individual differences in aggression (18, 73–75).

Another brain area that appears to play a significant role in aggressive behavior is the CeA, since impulsive aggression occurs when the amygdala (Amy) is overactivated with inadequate regulation from the PFC (47). The CeA, with its complex structure and extensive connections to other areas of the brain, plays a significant role in behavior (47, 49, 54). CeA has previously been reported to be more closely associated with predatory aggression than with territorial or hyperarousal aggression related to PWSI (49). Our data, demonstrating an increased number of c-Fos positive cells in the Amy and PFC of SHR/Izm rats, affirms that this rat strain is a suitable model for aggression in ADHD. The heightened neuronal activity in the Amy and PFC of SHR/Izm rats is consistent with a study that used functional magnetic resonance imaging in children with ADHD. The study found that higher emotional instability ratings correlated with stronger positive intrinsic functional connectivity between the amygdala and rostral anterior cingulate cortex (48).

Since aggressive behavior is related to Amy hyperactivity, a strategy to reduce this hyperactivity might decrease aggressive behavior. Interestingly, the effect of MSG on reducing attack behavior coincided with a decrease in c-Fos positive number of CeA. However, it should be noted that the reduction of CeA activity after MSG ingestion is detected under PWSI conditions where the CeA is in a hyperactive specific state. Since modulation of the CeA could be one of the potential mechanisms for reducing aggression levels in an ADHD rat model, it would be interesting to know which type of neuron or neurotransmitter in the CeA is affected by MSG ingestion. It was reported that social isolation-induced aggression increases glutamatergic activity by increasing AMPAR expression in the CeA (76, 77). Since the CeA mainly consists of inhibitory GABAergic cells, we hypothesized that MSG ingestion possibly stimulates the activity of inhibitory neurons in the CeA, thereby decreasing its total activity, especially excitatory neurons, under conditions that induce aggression (78). Further studies are required to understand how MSG ingestion modulates neuronal activity in the CeA and ultimately leads to behavioral changes.

It has been previously reported that MSG administration evoked c-Fos activity in the NTS (79, 80). Our results also showed that oral and intragastric administration of MSG increased c-Fos positive cells in the intermediate NTS (iNTS), confirming ascending viscero-sensory stimulation by MSG via the vagus nerve. In contrast, we found no c-Fos-positive cells in the rNTS (terminal of the tongue sensory afferent fiber of the glossopharyngeal nerve) after intragastric MSG administration. As glutamate receptors such as T1R1/T1R3, mGluR4, and mGluR1, are expressed on the epithelial mucosa in the stomach and intestine (81, 82), MSG can activate gut-brain signaling mediated by the vagus nerve that terminates in the iNTS, a part of the caudal NTS (cNTS) at the level of the area postrema (52). The important role of the vagus nerve in the effect of MSG was confirmed by vagotomy, which successfully diminished decreasing aggression and modulation of c-Fos activity in the iNTS and CeA. The gut-brain interaction in nutrient sensory transduction is mediated by two systems: one through an electrically excitable cell known as the enteroendocrine cell, and the other via an indirect system utilizing slow endocrine action of hormones such as CCK. Thus, the brain can perceive gut sensory cues through faster neuronal signaling mediated by the ‘Neuropod Cell,’ which connects the intestinal lumen to the brain stem in a single synapse (83). As the sugar stimulus from enteroendocrine cells in the intestinal lumen is transduced to vagal neurons using glutamate as a neurotransmitter, it raises an intriguing question: whether the MSG stimulus is also transmitted via Neuropod Cells, and which neurotransmitter facilitates the rapid transfer of sensory signals to vagal neurons upon MSG ingestion (84).

Interestingly, a bidirectional connection between the caudal NTS and the CeA has been reported (51, 54). The presence of CeA projection neurons from the cNTS in bregma levels −14.86 to −13.60, including at the level of AP (iNTS), has been identified in rats, suggesting an ascending efferent system from the cNTS to the CeA (53, 54). Consequently, iNTS activation by MSG via the vagus nerve potentially influences the activity of various brain regions involved in the control of both behavior and physiology (46). Moreover, our data showed that vagotomy successfully inhibited the effect of MSG in decreasing CeA hyperactivity in PWSI. Under normal conditions without inducing aggression (Figures 7A–E), our data showed that MSG increased c-Fos-positive cells in both the iNTS and CeA. Therefore, one possible pathway for the MSG effect based on our data is the projection of neuronal activation from the iNTS inhibiting hyperactivity in the CeA, possibly by the stimulation of inhibitory neurons in the CeA (46). However, we cannot exclude the possibility of indirect mechanisms through other areas of the brain associated with aggression, as the cNTS establishes a broad network involving multiple brain regions, not only the CeA, but also the PBL, PAG, PVH, LC, and BNST (53). A previous study showed that the administration of umami-rich dried bonito broth reduced aggressiveness, correlating with the densities of parvalbumin-immunoreactive neurons in the mPFC, amygdala, and hippocampus. This correlation indicates a potential indirect mechanism that is not mediated by the vagus nerve (85).

In this study, although MSG ingestion did not completely eliminate aggression, the significant decrease in strong aggressive behavior shows the possibility for a promising adjunctive therapy along with other strategies to manage aggression. It has been reported that daily intake of MSG in Europe and United States is 0.3–0.5 g/day while in Asia is 1.2–1.7 g/day (86). In this study, the human equivalent dose (HED) was estimated from the daily intake per kilogram body weight (kgBW/d) of rats, involving allometric scaling by multiplying with the Km ratio of 0.162 and adjusting for the high sensitivity of the taste receptor T1R1/T1R3 in humans by dividing by 20 (82, 87, 88). Based on these data, the MSG daily intake in this experiment is estimated to be equal to 22.55–26.98 mg/kgBW/d in humans (Supplementary Figure S1). This estimation is considered safe according to the population-acceptable daily intake (ADI) of MSG, which is proposed as 32 mg/kg BW/day by the European Food Safety Authority (EFSA), and the “No Observed Adverse Effect Level” dose is 3,200 mg/kg BW/day (89).

## 5 Conclusion

This study established improved experimental conditions to investigate the detailed brain mechanisms involved in reducing aggression induced by MSG. The resident-intruder test was used to observe aggressive behavior instead of the social-interaction test, and aggression was observed under red dim light during the subjective night. A comparison of isolation-induced aggression was performed in different rat strains, revealing that the ADHD rat model, SHR/Izm, is the most aggressive strain compared to Long-Evans, WKY/Izm, and SHR/Ezo. Immunostaining of c-Fos in aggression-related brain areas indicated that strong c-Fos expression in the mPFC, CeA, and LH may be linked to heightened aggression in SHR/Izm. Interestingly, the effect of MSG on decreasing aggression was confirmed by the resident-intruder test in SHR/Izm, possibly related to iNTS activation via the vagus nerve and modulation of CeA hyperactivity under conditions of PWSI-induced aggression. The effects of MSG on aggression and c-Fos expression in the iNTS and CeA were diminished by vagotomy. The effects of MSG stimulation on iNTS and CeA were also detected by direct single intragastric administration of MSG. The modulation of CeA activity is potentially linked to iNTS activation. Therefore, the iNTS-CeA projection, whether direct or indirect, could be a key mechanism in the effect of MSG on reducing aggressive behavior.

## Data availability statement

The original contributions presented in the study are included in the article/Supplementary material, further inquiries can be directed to the corresponding author. Requests to access the datasets should be directed to hhida@med.nagoya-cu.ac.jp.

## Ethics statement

The animal study was approved by the committee on animal experimentation of Nagoya City University Medical School. The study was conducted in accordance with the local legislation and institutional requirements.

## Author contributions

DM: Data curation, Formal analysis, Investigation, Methodology, Visualization, Writing – original draft. YN: Data curation, Formal analysis, Investigation, Methodology, Writing – original draft. SU: Data curation, Investigation, Methodology, Project administration, Writing – original draft. ST: Methodology, Writing – original draft. TS: Methodology, Validation, Writing – review & editing. NT: Methodology, Validation, Writing – review & editing. C-GJ: Validation, Writing – review & editing. HH: Conceptualization, Funding acquisition, Methodology, Project administration, Supervision, Validation, Writing – review & editing.

## Supplementary material

SUPPLEMENTARY FIGURE 1 Daily intake of MSG in experimental rats and estimation of the human equivalent dose of MSG. **(A)** The volume of drinking consumed by SHR/Izm in the MSG group (MSG; *n* = 12) and control group (dH_2_O; *n* = 12) was measured during five-week PWSI and MSG administration from P25 until P60. **(B)** Daily intake of MSG in the MSG group was calculated from the volume of drinking water containing 60 mM MSG consumed per day. **(C)** Body weight was measured during five-week PWSI and MSG administration period from P25 until P60. **(D)** The dose of MSG administered was calculated based on the daily intake of MSG per rat body weight. **(E)** The human equivalent dose (HED) was calculated from the daily intake per kilogram body weight of rats, involving allometric scaling by multiplying with the Km ratio of 0.162, and the adjusted HED was estimated by dividing 20 regarding the high sensitivity of the taste receptor T1R1/T1R3 in humans.
