# Supplementary figures and images for: Central amygdala is related to the reduction of aggressive behavior by monosodium glutamate ingestion during the period of development in an ADHD model rat
Source: Front Nutr. 2024 May 3;11:1356189. doi: 10.3389/fnut.2024.1356189 (PMC11099272; doi:10.3389/fnut.2024.1356189)

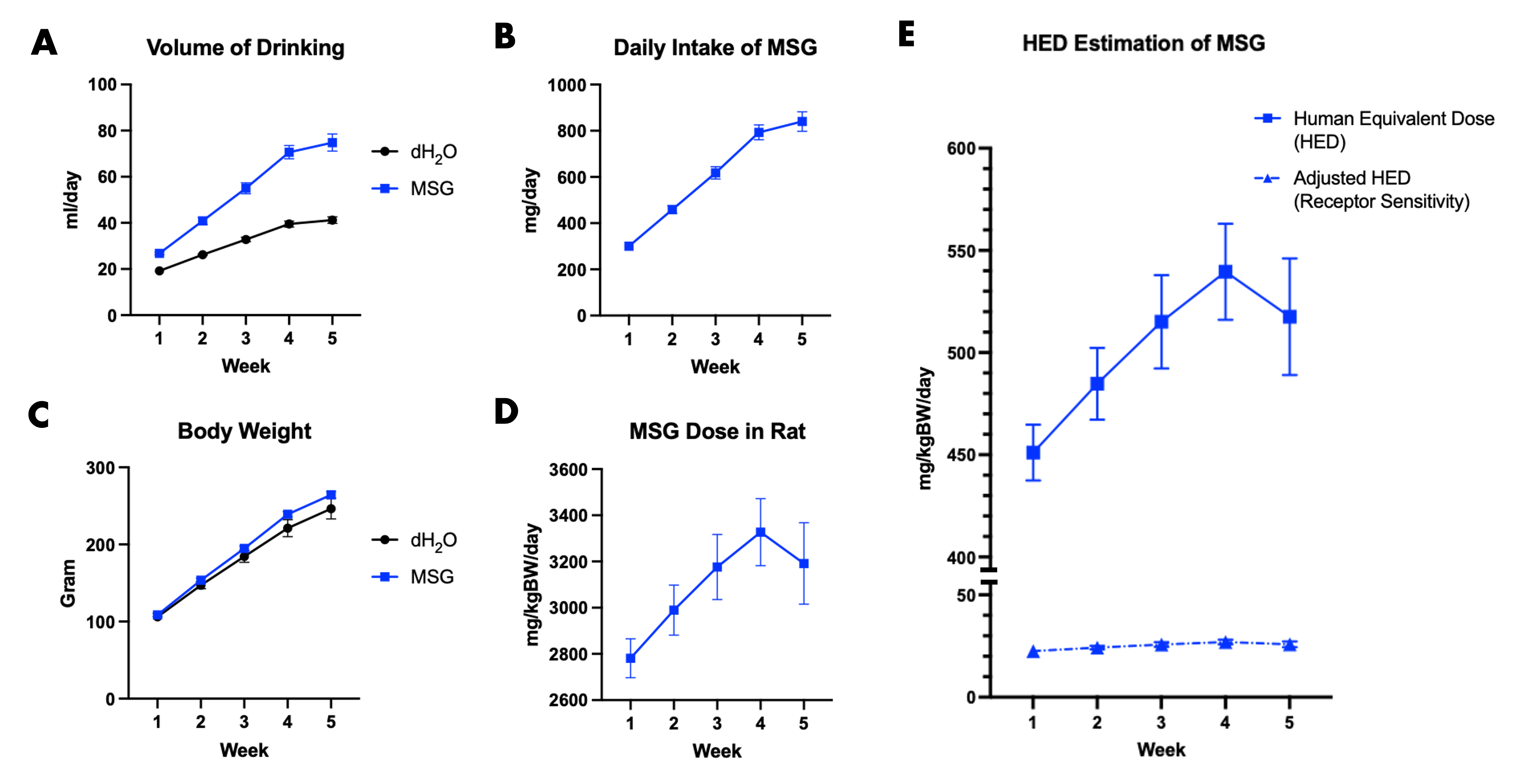

Supplement: SUPPLEMENTARY FIGURE 1 — Daily intake of MSG in experimental rats and estimation of the human equivalent dose of MSG. (A) The volume of drinking consumed by SHR/Izm in the MSG group (MSG; n = 12) and control group (dH2O; n = 12) was measured during five-week PWSI and MSG administration from P25 until P60. (B) Daily intake of MSG in the MSG group was calculated from the volume of drinking water containing 60 mM MSG consumed per day. (C) Body weight was measured during five-week PWSI and MSG administration period from P25 until P60. (D) The dose of MSG administered was calculated based on the daily intake of MSG per rat body weight. (E) The human equivalent dose (HED) was calculated from the daily intake per kilogram body weight of rats, involving allometric scaling by multiplying with the Km ratio of 0.162, and the adjusted HED was estimated by dividing 20 regarding the high sensitivity of the taste receptor T1R1/T1R3 in humans. [file Image_1.TIF]
